# Hercynine, Ergothioneine and Redox State in Stallion’s Seminal Plasma

**DOI:** 10.3390/antiox9090855

**Published:** 2020-09-13

**Authors:** Salvatore Sotgia, Andrea Taras, Angelo Zinellu, Raffaele Cherchi, Arduino A Mangoni, Ciriaco Carru, Luisa Bogliolo

**Affiliations:** 1 Department of Biomedical Sciences, School of Medicine, University of Sassari, 07100 Sassari, Italy; azinellu@uniss.it (A.Z.); carru@uniss.it (C.C.); 2 Agricultural Research Agency of Sardinia (AGRIS)—Research Service for Equine Production and Reproduction, 07014 Ozieri, Italy; andreataras@yahoo.it (A.T.); ilex283@gmail.com (R.C.); 3 Discipline of Clinical Pharmacology, College of Medicine and Public Health, Flinders University and Flinders Medical Centre, Adelaide 5001, Australia; arduino.mangoni@flinders.edu.au; 4 Quality Control Unit, University Hospital Sassari (AOUSS), 07100 Sassari, Italy; 5 Department of Veterinary Medicine, University of Sassari, 07100 Sassari, Italy; luis@uniss.it

**Keywords:** oxidative stress, seminal plasma, redox state, aminothiols, aminothione

## Abstract

The dependence of a stallion’s spermatozoa on oxidative phosphorylation for energy requirements results in an unconventional relationship between reactive oxygen species (ROS) production and fertility. In such a scenario, antioxidant activity must be finely controlled and not affect the essential functions of ROS. Some in vivo evidence suggests that the naturally occurring antioxidant ergothioneine (ERT) interferes with the critical roles of ROS/reactive nitrogen species (RNS) in pro-oxidant states but not in healthy tissues. The measurement of ERT in seminal plasma collected from 14 stallions (five Anglo-Arab, five Sella Italiano and four Thoroughbreds of which three are Arabian and one English) aged 16 ± 6 years (range 6–25 years) confirms that ERT is present at high concentrations in this biological fluid, between 16.80 and 971.48 µmol/L. Although the presence of high ERT concentrations in the seminal plasma of a stallion has long been known, its exact biological role is uncertain. This might be due to the peculiar antioxidant cycle of ERT, specifically its rapid recovery, which potentially masks concentration fluctuations and, therefore, the extent of its physiological effects. The measurement of the ERT precursor and redox metabolite hercynine (ERY) may overcome such issues, as ERY does not undergo regeneration processes. ERY was detectable and measurable in the seminal plasma of all stallions at a median concentration of 7.50 (IQR 15.26) nmol/L. The analysis of the association between the ERT and ERY, as well as with other established antioxidants such as glutathione and cysteine, suggests that ERT may play a major role in the antioxidant machinery of seminal plasma, and that ERY might serve as a new combined marker of oxidative stress and semen quality.

## 1. Introduction

The relationship between stallions’ fertility and oxidative stress is more complex than other species. This appears to be linked to the specific metabolic pathway in stallion spermatozoa to meet energy demands, which, unlike most species, is more dependent on mitochondrial ATP production by oxidative phosphorylation (OXPHOS) than glycolysis [1,2,3,4]. The former is more efficient in producing ATP and promoting higher sperm motility leading, however, to increased production of reactive oxygen species (ROS) [5]. In particular, about 1–3% of O_2_ reduced in the mitochondria during OXPHOS, forms the superoxide radical [5]. The high production of ROS, therefore, appears to be physiological in stallions and contrasts with the traditional view that ROS indicate the presence of nonviable or poor quality spermatozoa [6]. This results in a positive, and counterintuitive, correlation between fertility and oxidative stress [2]. Spermatozoa, however, are inherently inefficient, when compared to somatic cells, in protecting against the potentially detrimental effects of ROS [7]. ROS homeostasis is maintained by different components with the antioxidants function in the seminal plasma [8], a complex mixture of secretions produced by the testes, epididymis and the accessory sex glands [9].

Among these components, there has been an increasing interest toward ergothioneine (ERT, 2-mercaptohistidine trimethylbetaine), as several in vitro studies highlighted its antioxidant effects [10]. ERT is widely distributed in the blood and tissues of vertebrates, although its biosynthesis is limited to certain bacteria, such as mycobacteria and cyanobacteria, and some fungi, including edible mushrooms [11,12,13]. In higher animals, ERT is supplied with food and primarily stored as an intracellular constituent [11,14]. However, in some animals, e.g., the boar, hedgehog, donkey and horse, ERT also occurs as an extracellular component of semen where it reaches high concentrations [15]. Despite the presence of ERT in the semen of different animals, the primary source may differ [16]. In stallions, the ampulla of the vas deferens is the primary secretor of ERT, with reported local concentrations between 1.7 [16] and 2.8 [17] mmol/L. The tautomeric equilibrium of ERT between thiol and thione forms, with the latter predominant at physiological pH, provides ERT with peculiar stability and reactivity compared to other naturally occurring alkylthiols such as glutathione (GSH) and cysteine (Cys) [18]. For example, at physiological pH ERT does not auto-oxidize [19] nor stimulates lipid peroxidation in the presence of ferric ions [18]. Moreover, ERT oxidizes to the corresponding disulfide (ESSE) [20]. The latter, being unstable at physiological pH, undergoes a spontaneous disproportionation rather than an enzymatic-driven reconversion to the reduced form, as observed with other disulfides. From this disproportionation, hercynine (ERY; N,N,N-trimethyl-histidine), the main precursor of ERT biosynthesis, and ERT are generated and back-formed, respectively [20,21]. Without any enzymatic catalysis, the spontaneous decomposition occurring at a physiological pH of 2 mol of ESSE leads to the production of 1 mol of ERY and the reformation of 3 mol of ERT [20,21]. This peculiar antioxidant mechanism seems to confer an advantage in vivo as ERT depletion is relatively slow [22]. Furthermore, its prompt recovery can start a new redox cycle faster than other antioxidants such as GSH [22].

In view of its antioxidant properties, some studies have investigated the effects of ERT as an antioxidant freezing additive extender to improve the cryopreservation process of semen [23] or the in vitro fertilization and embryonic development of ovine oocytes [24]. ERT concentrations have also been investigated in the amniotic fluid of pregnant sheep after natural mating or transfer of vitrified/thawed in vitro produced embryos [25] and cow milk [26], as well as in the follicular fluid of different farm animals [27]. Moreover, earlier studies from the late 1950s reported that in a stallion’s seminal fluid an inverse relationship exists between fertility and the concentration of the non-protein thiol fraction, mainly represented by ERT [28,29]. A decline in the ability to maintain motility in spermatozoa was observed for concentrations exceeding 10 µg of non-protein thiol/mL, and infertility for levels above 20 µg of non-protein thiol/mL [29]. However, the nature of this relationship has remained unclear. One possible reason is the fast recovery of ERT following oxidation, which could mask concentration fluctuations in biological fluids and, therefore, prevent the identification of its exact biological effects. By contrast, the measurement of ERY, both the main precursor of ERT biosynthesis in producing organisms and its major oxidative metabolite, might offer several advantages over ERT as ERY does not undergo regeneration processes. However, despite the significant amount of research on a stallion’s semen, no study has investigated the redox connection between ERT and ERY and the role of ROS in stallion fertility. We investigated the usefulness of ERY assessment to ascertain the biological role of ERT and the contribution of ERY/ERT redox coupling in explaining the unexpected relationship between oxidative stress and fertility observed in stallions. Furthermore, we assessed the role of ERT and ERY as markers of semen quality.

## 2. Materials and Methods

### 2.1. Animals, Semen and Seminal Plasma Samplings

Fourteen stallions (5 Anglo-Arab, 5 Sella Italiano and 4 Thoroughbreds of which 3 were Arabian and 1 English) aged 16 ± 6 years (range 6–25 years) were studied. The horses were in good condition, kept in the same farm in individual stalls bedded with straw and with freely available water, and fed with hay ad libitum integrated with a balanced ration of commercial horse food. Movement of stallions was provided by a carousel, where they walked one hour a day. Ejaculates were regularly collected from February to June 2012 using a lubricated prewarmed artificial vagina (INRA model) equipped with an in-line gel filter after stimulation of stallions by mare situated close to the breeding phantom. To reduce extragonadal sperm reserves of the cauda epididymis and vas deferens, including the ampulla, for two days, one day apart from each other, the semen was withdrawn and discarded from each stallion. Thereafter, ejaculates were collected for four days, one day apart from each other, and the volume of each ejaculate recorded. An aliquot of each gel-free portion was then diluted 1:1 with a warmed (37 °C) milk extract-based extender (INRA 96^®^; IMV, Maple Grove, MN, USA) and analyzed using a computer-assisted sperm analyzer (Sperm Class Analyzer^®^; Microptic, Barcelona, Spain) equipped with SpermVision software (Minitube, Tiefenbach, Germany) and the microscope Olympus BX 51 (Olympus, Japan) for sperm motion characteristics, including percentages of total motile spermatozoa. For this purpose, diluted samples were placed into a Makler counting chamber (Sefi-Medical Instruments, Haifa, Israel) with a volume of 10 μL heated to 37 °C for each analysis. A 10 mL aliquot of gel-free ejaculates maintained on ice was centrifuged twice at 1200× *g* for 10 min at 4 °C to obtain the seminal plasma that was stored as 2 mL aliquots at −20 °C until use. All experimental procedures were carried out at AGRIS (Agricultural Research Agency of Sardinia, Ozieri, Italy), which meet the requirements of the European Union for Scientific Procedure Establishments. The procedures followed ethical guidelines for care and use of animals for research (European Union Directive 2010/63/UE for animal experiments) and were approved by the Animal Care and Use Committee of the University of Sassari and AGRIS.

### 2.2. Quantification of Ergothioneine (ERT) in Seminal Plasma

ERT was measured according to the method developed by Sotgia et al. (2013) [30] with some modifications. Briefly, seminal plasma was diluted 1/5 with ultrapure water (Milli-Q grade) then centrifuged at 17,000× *g* for 10 min at room temperature. After centrifugation, 50 µL of a solution containing 770 µmol/L of 5-iodoacetamidofluorescein in 150 mmol/L of sodium phosphate tribasic dodecahydrate buffer at pH 13 were added to 150 µL of supernatant. After vigorous vortex-mixing, the reaction mixture was left in a light-protected area for 30 min at room temperature. Finally, samples were analyzed by a P/ACE MDQ capillary electrophoresis apparatus equipped with a laser-induced fluorescence detector (LIF; Beckman-Coulter Italia, Milan, Italy). The analysis was performed in an uncoated fused silica capillary, 50 µm id and 60 cm length (50 cm to the detection window), injecting 3.87 nL of sample (0.5 psi × 5 s). The separation was carried out by using a 20 mmol/L sodium phosphate tribasic dodecahydrate solution as a running buffer, 15 °C and 30 kV (70 µA) at normal polarity. Derivatized samples were held at 10 °C in the autosampler and detected by a LIF detector.

### 2.3. Quantification of Hercynine (ERY) in Seminal Plasma

ERY was measured according to the assay described by Sotgia et al. (2018) [31] with some modifications. Briefly, a 200 µL-volume of ultrapure water milli-Q grade containing a deuterated version of ERY used as an internal standard at a concentration of a 100 nmol/L was added to 200 µL of seminal plasma. After vortex-mixing and centrifugation at 17,000× *g* for 10 min at room temperature, a 20 µL volume of a 100 mmol/L sodium phosphate dibasic heptahydrate buffer and 40 µL of a 33 mmol/L diethylpyrocarbonate solution were added to a 120 µL volume of supernatant. The reaction mixture was mixed thoroughly by vigorous vortex-mixing then allowed to stand at room temperature for 5 min before analysis by LC–MS/MS. The latter was a Waters system model Acquity UPLC equipped with a Waters Acquity UPLC tandem quadrupole mass spectrometer (Waters Italia, Milan, Italy). The separation was achieved on a 100 mm × 4.6 mm Zorbax Eclipse Plus C18 3.5 µm column by using a mixture of aqueous of 0.1% v/v formic acid and ACN (95:5) as a mobile phase, isocratically delivered at a flowrate of 0.5 mL min^-1^. Column effluents were monitored by mass spectrometry multiple reaction monitoring mode (MRM). Mass detection was accomplished in positive ion mode by MRM using precursor–product ion transitions *m/z* 270.28→95 and 273.21→95 for ERY and the internal standard, respectively. Cone voltage, collision voltage and dwell times were 28 V, 27 V and 0.1 s for ERY, and 35 V, 25 V and 0.1 s for the internal standard, respectively.

### 2.4. Quantification of Glutathione (GSH) and Cysteine (Cys) in Seminal Plasma

The low-molecular-weight thiols GSH and Cys were measured as described by Carru et al. (2004) [32]. Briefly, to reduce disulfide bounds, a 20 µL volume of 10% *v/v* tri-n-butylphosphine in dimethylformamide was added to 200 µL of seminal plasma. After vortex-mixing, 200 µL of 6% w/v 5-sulphosalicylic acid was added then the mixture was centrifuged at 17,000× *g* for 5 min. A 100 µL volume of 300 mmol/L sodium phosphate dodecahydrate at pH 12.5 and 25 µL of 4.1 mmol/L 5-iodoacetamidofluorescein were added to 100 µL of supernatant. After vortex-mixing, the reaction mixture was left in a light-protected area for 15 min at room temperature before 100-fold dilution with ultrapure water (Milli-Q grad) and analysis by a P/ACE MDQ capillary electrophoresis system equipped with a laser-induced fluorescence detector (LIF; Beckman-Coulter Italia, Milan, Italy). The analysis was performed in an uncoated fused silica capillary, 75 µm id, and 57 cm length (50 cm to the detection window), injecting 14 nL of sample (0.5 psi × 2 s). The separation was carried out using a background electrolyte at a pH of 11 containing 5 mmol/L of sodium phosphate tribasic dodecahydrate, 4 mmol/L of boric acid and 75 mmol/L of N-methyl-D-glucamine. The electrophoretic conditions, 28 kV, 70 mA and normal polarity, were reached in 20 s then held at a constant voltage for 5 min.

### 2.5. Quantification of Free Thiol Groups in Seminal Plasma (DTNB)

The concentration of total thiol groups in seminal plasma (-SH) was measured by the method originally described by Ellman [33]. In this colorimetric assay, the water-soluble Ellman’s reagent (DTNB, 5,5′-dithio-bis(2-nitrobenzoic acid)) interacts with thiols becoming the 5-mercapto-2-nitrobenzoic acid, a measurable highly yellow-colored anion with an absorption maximum peak at 412 nm. DTNB is sensitive to the content of the free thiols in proteins, peptides and tissues, especially to cysteine residues. For the analysis, DTNB was prepared as a stock solution at a concentration of 2 mmol/L in 50 mmol/L sodium acetate. A working solution of DTNB was obtained by mixing 50 µL of the stock DTNB solution to 100 µL of a 1 M Tris solution at a pH of 8 and 840 µL of ultrapure water (Milli-Q grade). After careful vortex-mixing, 10 µL of the sample was added to 990 µL of the DTNB working solution then the mixture was left to incubate for 5 min. The absorbance of the samples was then read with a spectrophotometer and quantitative analysis was obtained using a standard calibration curve for the -SH group (acetylcysteine) starting at 10 µmol/L.

### 2.6. Trolox Equivalent Antioxidant Capacity Assay (TEAC)

The total antioxidant activity of seminal plasma was measured through the comparison with the antioxidant capacity of Trolox, a water-soluble analogs of vitamin E. Trolox equivalency was assessed using the ABTS decolorization assay as described by Re et al. (1999) [34]. Briefly, to produce ABTS radical cation (ABTS^•+^), the ABTS stock solution at a concentration of 7 mmol/L in 20 mmol/L sodium acetate buffer at pH 6.5 was mixed with 2.45 mmol/L potassium persulfate. Then, the mixture was allowed to stand in the dark at room temperature for 12–16 h. Before use, the ABTS^•+^ solution was diluted with PBS buffer (pH 7.4) to produce an absorbance of 0.70 (±0.02) at 734 nm and equilibrated at 30 °C. Seminal plasma was diluted with ultrapure water (Milli-Q grade) to give an inhibition of the blank absorbance between 20 and 80%. A 1 mL volume of diluted ABTS^•+^ solution (A_734 nm_ = 0.7 ± 0.02) was added to 10 µL of the diluted sample or the Trolox standards (final concentration 0–15 mmol/L). The absorbance was taken at 30 °C exactly 1 min after initial mixing and up to 6 min. The percentage inhibition of absorbance at 734 nm (I) was calculated by the following formula I = [1 − (A_f_/A_0_)] × 100 where A_0_ is the absorbance of the radical cation solution before addition of the sample/standard antioxidant and A_f_ is the absorbance after addition of the sample/standard antioxidant.

### 2.7. Statistical Analysis

Data were assessed for normality using the Kolmogorov–Smirnov test and presented as either range, mean ± standard deviation (SD) or median and interquartile range (IQR) computed as the difference between the 75th and 25th percentiles, as appropriate. Intra- and interindividual variation were expressed as the percent coefficient of variation (%CV). Differences between groups were assessed by a repeated-measures analysis of variance (ANOVA) or Friedman test as appropriate. As the total correlation mixing both between- and within-subject correlations can be misleading [35] associations were computed as the repeated-measures correlation coefficient (Rm) as described by Bland and Altman [36] and Bakdash and Marusich [37]. A two-sided *p* value of 0.05 was chosen as the cut-off for statistical significance. Statistical analyses were performed using MedCalc Statistical Software for Windows, version 17.5.5, 64 bit (MedCalc Software bvba, Ostend, Belgium), Small Stata for Windows, version 14.1, 64 bit (StataCorp LP, College Station, TX, USA) and RStudio for Windows, version 1.2.5033 (RStudio Inc., Boston, MA, USA).

## 3. Results

Table 1 displays the principal parameters considered in the study.

**Figure 1:**
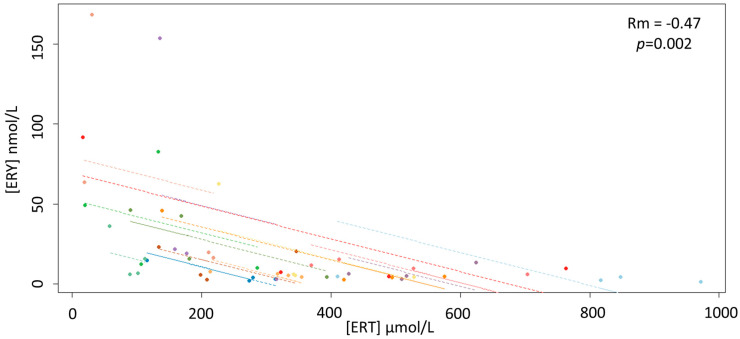
Scatterplots of the linear relationship between the concentrations of ERY and ERT.

ERT concentrations were normally distributed over the four withdrawals with a global mean concentration of 319.56 ± 222.75 µmol/L (range 16.80–971.48 µmol/L) resulting in a %CV of 70%. The intraindividual variation in the four samplings ranged between 16 and 92% while the interindividual variations ranged between 64% on the fourth day and 73% of the second day of collection. Repeated measures ANOVA showed no significant temporal difference in ERT concentrations as well as no linear or other types of trends. Unlike ERT, ERY was skewed across the four samplings with an intraindividual %CV that ranged between 24 (5.87 ± 1.42 nmol/L) and 149% (28.41 ± 42.28 nmol/L), and a global median value of 7.50 (IQR 15.26) nmol/L. The interindividual %CV ranged between 130 and 188% although the Friedman test did not indicate significant differences (*p* = 0.40) among the median values within any of the four groups: 6.31 (IQR 16.10), 6.41 (IQR 11.21), 5.33 (IQR 15.43) and 11.54 (IQR 12.66) nmol/L from the first group to the last, respectively. Median concentrations of GSH showed a trend to decrease from the first to the last day of samplings with values, respectively, of 1.71 (IQR 1.29), 1.28 (IQR 0.98), 1.11 (IQR 0.44) and 0.98 (IQR 1.05) µmol/L. The Friedman test showed a significant temporal difference between GSH concentrations (*p* = 0.02), in particular between the first withdrawal with the third and fourth. The total median concentration of Cys was 32.32 (IQR 29.60) µmol/L with a significant temporal decrease from the first to the last day of samplings (Friedman test, *p* = 0.02), in particular between the first withdrawal (40.24 (IQR 34.08) µmol/L) and the second (32.47 (IQR 22.09) µmol/L), third (25.38 (IQR 33.10) µmol/L) and fourth (29.90 (IQR 35.65) µmol/L). Among samplings, no significant differences in the concentrations of free thiol groups (DTNB) were observed by the Friedman test (*p* = 0.83), as well as between the Trolox equivalent antioxidant capacity (TEAC) values (*p* = 0.052). The interindividual spermatozoa motility medians from the first samplings to the last were 81%, 86%, 83% and 87% with a %CV range between 17 and 28%, without significant temporal differences (Friedman test, *p* = 0.33). The intraindividual spermatozoa motility variations were wider, ranging between 0 and 59%. As shown in Figure 1, the repeated-measures correlation analysis showed a significant negative correlation between ERT and ERY concentrations (Rm = −0.47; 95% confidence interval −0.68, −0.18; *p* = 0.002).

**Figure 2:**
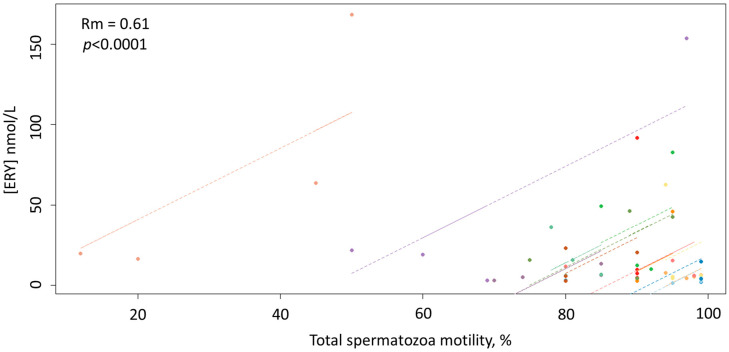
Scatterplots of the linear relationship between the concentrations of ERY and the total spermatozoa motility.

As displayed in Figure 2, the repeated-measures correlation analysis also showed a significant positive correlation between ERY concentrations and total spermatozoa motility (Rm = 0.61; 95% confidence interval 0.37, 0.77; *p* < 0.0001).

**Figure 3:**
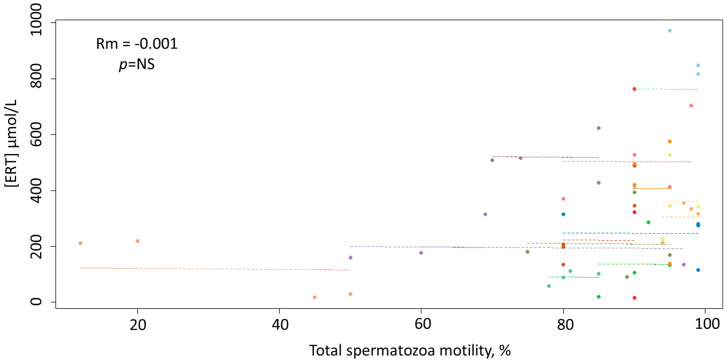
Scatterplots of the linear relationship between the concentrations of ERT and the total spermatozoa motility.

As depicted in Figure 3, unlike ERY, ERT concentrations did not correlate significantly with the total spermatozoa motility (*p* = 0.94).

ERT concentrations correlated significantly with TEAC (Rm = 0.56; 95% confidence interval 0.30, 0.74; *p* < 0.0001), while no significant correlation was observed with DTNB (*p* = 0.98), GSH (*p* = 0.78) and Cys (*p* = 0.08). ERY concentrations showed a significant negative correlation with TEAC (Rm = −0.33; 95% confidence interval −0.58, −0.02; *p* = 0.03), GSH (Rm = −0.32; 95% confidence interval −0.57, −0.02; *p* = 0.03) and Cys (Rm = −0.33; 95% confidence interval −0.58, −0.02; *p* = 0.03), while no significant correlation was observed with DTNB (*p* = 0.41). GSH and Cys correlated positively and significantly (Rm = 0.32; 95% confidence interval 0.02, 0.58; *p* = 0.03) and both were significantly and negatively correlated with the total spermatozoa motility, Rm = −0.34; 95% confidence interval -0.60, −0.04; *p* < 0.02 and Rm = −0.40; 95% confidence interval −0.63, −0.11; *p* < 0.008, respectively. No correlation was observed between DTNB and GSH (*p* = 0.32) or Cys (*p* = 0.53) and between TEAC and GSH (*p* = 0.79). However, a significant correlation was observed between TEAC and Cys (Rm = 0.37; 95% confidence interval 0.069, 0.61; *p* = 0.01).

## 4. Discussion

The average median concentrations of GSH and Cys measured in this study were, respectively, around 1 and 30 µmol/L. The observed GSH concentrations are in line with the low levels measured by LC–MS/MS methods [15], thus confirming that its amount in the seminal plasma of a stallion is significantly lower than previously reported values measured with colorimetric assays [15]. It should also be noted that the concentrations of Cys and GSH reported herein were computed as the sum of their reduced-free, oxidized-free and protein-bound forms [32]. Therefore, the concentrations of the reduced-free forms, the only ones with antioxidant effects, could be even lower. The lack of correlation between Cys and GSH with DTNB, a chemical that reacts only with free -SH groups, would support this hypothesis as the probable result of a negligible contribution of their reduced-free forms to the whole free thiol groups reacting with DTNB. GSH and Cys showed a similar trend to decrease from the first to the last sampling and a positive and significant correlation (Rm = 0.32; 95% confidence interval 0.02, 0.58; *p* = 0.03). Such an association was not surprising as Cys is known to be a precursor in the biosynthesis of GSH. Thus, the decrease of GSH from one withdrawal to another could be due to the reduced availability of Cys for the synthesis. Neither Cys nor GSH correlated with ERT, and there was also no correlation between ERT and DTNB. This suggests that in the seminal plasma of a stallion there is not a functional link between ERT and the other two aminothiols, as well as with the free thiol groups of the proteins or of other low-molecular-weight thiols. Moreover, the absence of association with DTNB also highlights that ERT does not contribute to the total free thiol groups. Unlike other amino thiols, this is explainable in the light of the predominant form of ERT as thione instead of thiol, which prevents the coupling with DTNB.

Consistently with earlier observations [15], the average concentration of ERT in the seminal plasma of a stallion in this study was 319.56 ± 222.75 µmol/L (range 16.80–971.48 µmol). The presence of high amounts of ERT in this biological fluid has long been known although the reason is still unclear. The recent change of the long-standing paradigm that in stallion ROS are typically detrimental for spermatozoa function may offer an explanation. In a context where oxidative stress is due to redox deregulation rather than overproduction of ROS, in fact, the activity of antioxidants must be finely controlled while remaining at the same ready for action under specific circumstances. In this sense, some in vivo evidence shows that ERT does not interfere with the critical roles of ROS/RNS in healthy tissues and that its action is effective only when oxidative stress becomes intensive [38]. Thus, the significant positive correlation between ERT and TEAC (Rm = 0.56; 95% confidence interval 0.30, 0.74; *p* < 0.0001) suggests that ERT plays an important role in the antioxidant system of the seminal plasma of a stallion. Conversely, the lack of association between TEAC and GSH along with the weak one with Cys further supports the view that the content of their reduced-free forms in seminal plasma is low and, therefore, also the extent of their antioxidant action. However, their negative correlation with the total motility of spermatozoa, suggests that some biological activity is still retained by GSH and Cys, which, interestingly, may be harmful for spermatozoa functionality. This finding is consistent with similar results regarding other antioxidants such as ascorbic acid [39] and fits with the dependence of stallion sperm on OXPHOS for energy requirements reported by other authors [1,2,3,4]. In other words, the negative correlations between GSH and Cys and sperm motility would confirm the presence of the already reported paradoxical relationship between stallion fertility and oxidative stress [2].

Although the lack of any correlation between ERT and sperm motility is of difficult interpretation, given the antioxidant role suggested by the data, the peculiar redox cycle of ERT may account for this apparent discrepancy as it involves a fast recovery of ERT that potentially could hide concentration fluctuations that are linked to its biological activity [31,40]. Therefore, the overall ERT measurement might not discriminate between the ERT regenerated following oxidation and the whole ERT. As proposed in our previous studies [31,40], ERY measurement, instead of ERT alone, may overcome this issue, as this oxidative metabolite of ERT does not undergo regeneration processes [21,22]. In our study, ERY concentrations in stallion seminal plasma (global median value of 7.50 (IQR 15.26) nmol/L) were much lower and exhibited significant variability when compared to ERT. However, as expected for a compound formed following ERT oxidation, ERY was negatively correlated with ERT (Rm = −0.47; 95% confidence interval −0.68, −0.18; *p* = 0.002). Additional support for this proposition comes from the negative correlations observed between ERY and TEAC and the antioxidants Cys and GSH, which further indicate that ERY is an oxidative metabolite.

Notably, unlike ERT, ERY showed a strong and positive correlation with the total spermatozoa motility (Rm = 0.61; 95% confidence interval 0.37, 0.77; *p* < 0.0001). Taken together, these data provide evidence that ERY may be used to study the involvement of ERT in redox reactions, as the former is less likely to be affected by the peculiar redox cycle of ERT. Interestingly, considering that motility of spermatozoa and its maintenance are accepted criteria for the partial evaluation of the potential fertility of any given semen sample [29], the finding that this parameter closely correlates with the concentration of ERY, suggests that ERY might serve as a new marker of oxidative stress and, pending further studies, the quality of semen in the stallion.

## 5. Conclusions

To our knowledge, this is the first study to report a thorough investigation of the concentrations of ERY in the seminal plasma of a stallion. ERY may be useful to evaluate the extent of the biological activity of ERT and the latter seems to play an important antioxidant role in seminal plasma. Moreover, the close association between ERY and sperm motility supports the role of ERY as an oxidative metabolite and the notion that ROS are essential regulators of sperm function. Despite the preliminary nature of the reported results and the need for additional investigations involving other parameters characterizing spermatozoa and fertilization rate in vivo, ERY might represent a novel combined marker of oxidative stress and, in this role, of semen quality in the stallion. This could be of considerable interest in the equine industry as the recent findings on the relationship between stallions’ fertility and oxidative stress call for the identification of new markers for the assessment of the quality and fertility of stallion ejaculates.

## Figures and Tables

**Table 1:** Parameters measured in the study. Values are the mean±SD or median (interquartile range (IQR)) of the levels reached during the four withdrawals.

	Withdrawal #1	Withdrawal #2	Withdrawal #3	Withdrawal #4
ERT µmol/L ± SD	234.59 ± 156.13	310.61 ± 225.76	369.91 ± 260.70	363.01 ± 232.34
ERY nmol/L (IQR)	6.31 (16.10)	6.41 (11.21)	5.33 (15.43)	11.54 (12.66)
GSH µmol/L (IQR)	1.71 (1.29)	1.28 (0.99)	1.11 (0.44)	0.98 (1.05)
Cys µmol/L (IQR)	40.23 (34.08)	32.47 (22.09)	25.38 (33.09)	29.90 (35.65)
DTNB µmol/L (IQR)	3.36 (1.24)	4.20 (3.01)	3.18 (2.09)	3.63 (1.27)
TEAC μmolTE/100 g (IQR)	7.46 (1.36)	6.33 (2.97)	8.19 (5.98)	6.10 (4.59)
Sperm Volume mL ± SD	25 ± 12	32 ± 13	30 ± 11	33 ± 14
Sperm Concentration million per mL (IQR)	360 (288)	358 (169)	415 (231)	402 (169)
Total Motility % (IQR)	83 (10)	93 (17)	90 (15)	91 (5)
